# Pain and Coping Strategies as Determinants of Malnutrition Risk in Lung Cancer Patients: A Cross-Sectional Study

**DOI:** 10.3390/nu16142193

**Published:** 2024-07-10

**Authors:** Jacek Polański, Wojciech Tański, Krzysztof Dudek, Beata Jankowska-Polańska

**Affiliations:** 1 Department of Internal and Occupational Diseases, Hypertension and Clinical Oncology, Wroclaw Medical University, 50-556 Wrocław, Poland; 2 Faculty of Medicine, Wrocław University of Science and Technology, 50-376 Wrocław, Poland; wojciech.tanski@pwr.edu.pl (W.T.); bpolanska@4wsk.pl (B.J.-P.); 3 Department of Internal Medicine, 4th Military Clinical Hospital, 50-981 Wrocław, Poland; 4 Faculty of Mechanical Engineering, Wroclaw University of Science and Technology, 50-370 Wrocław, Poland; krzysztof.dudek@pwr.edu.pl; 5 Centre for Research and Innovation, 4th Military Clinical Hospital, 50-981 Wrocław, Poland

**Keywords:** lung cancer, malnutrition, pain level, pain control, coping strategies

## Abstract

Progressive cachexia and malnutrition severely impact the physical and mental condition of cancer patients. Pain is a prognostic factor for shorter survival in cancer patients, and coping strategies are crucial for adapting to treatment and dietary regimens. This study assessed pain levels, pain-related beliefs, and coping strategies as factors increasing malnutrition risk in 257 lung cancer patients. Sociodemographic and clinical data were collected from medical records. The Mini Nutritional Assessment (MNA), Visual Analog Scale (VAS), Beliefs about Pain Control Questionnaire (BPCQ), and Coping Strategies Questionnaire (CSQ) were used. Overall, 42.8% of patients were at risk of malnutrition, and 17.5% were malnourished. Nutritional status negatively correlated with CSQ domains: reinterpretation of pain (RP: rho = −0.194; *p* = 0.002), catastrophizing (CP: rho = −0.414; *p* = 0.001), ignoring pain (IP: rho = −0.198; *p* = 0.001), praying/hoping (PH: rho = −0.253; *p* < 0.001), and coping self-statements (CS: rho = −0.172; *p* = 0.006); and BPCQ domains: the power of doctors (PD: rho = −0.196; *p* = 0.002) and VAS (rho = −0.451; *p* < 0.001). Nutritional status positively correlated with CSQ domains: pain control (PC: rho = 0.499; *p* < 0.001) and the ability to reduce pain (AR: rho = 0.512; *p* < 0.001). In multivariate regression analysis, a better nutritional status was associated with a younger age (β = −0.094; *p* < 0.001), non-small-cell lung cancer (NSCLC) (β = 1.218; *p* = 0.037), a greater ability to reduce pain (CSQ-AR) (β = 0.901; *p* < 0.001), lower catastrophizing (CSQ-CP) (β = −0.165; *p* = 0.001), and lower pain perceived (VAS) (β = 0.639; *p* < 0.001). Statistical analyses included Spearman’s correlation and multivariate regression with a significance level of *p* < 0.05. Patients with a normal nutritional status had reduced doctor involvement in pain control, less frequent negative coping strategies, and more common positive coping strategies. A normal nutritional status correlates with lower perceived pain. A better nutritional status is linked to a younger age, NSCLC, lower pain levels, greater pain reduction ability, and lower scores in pain catastrophizing.

## 1. Introduction

Lung cancer is the most common cause of cancer-related death, and the prognosis depends mainly on the stage at the time of diagnosis [1]. Other factors adversely affecting prognosis include the histological type of cancer, age, gender, mental status, and nutritional status. Malnutrition and even cachexia are prevalent in patients with advanced cancer [2]. A negative protein and energy balance is crucial in malnutrition and cancer cachexia. It results from the patient’s increased metabolic rate, decreased food intake, and metabolic disorders caused by strenuous treatment [3]. In addition to an insufficient calorie intake and impaired metabolism, the nutritional status of cancer patients is affected by fatigue, mood disorders, and treatment-related toxicity [4].

Malnutrition leads to the loss of muscle mass and a systemic inflammatory response characterized by leukocytosis and high levels of acute phase proteins [4]. It is indicated that malnutrition is associated with a worse response to treatment, poorer absorption of drugs, a higher risk of complications, an unfavorable prognosis, an increased frequency of hospitalizations, and a high risk of death [5]. Progressive cachexia and malnutrition severely affect the condition of the internal organs and muscles, limiting physical and mental activity, leading to reduced daily functioning and a deterioration in the quality of life [6].

Both the diagnosis of cancer and the resulting physical and mental challenges often lead to mood disorders, stress, and depression. These conditions increase the risk of malnutrition, which further exacerbates mood disorders and physical limitations [7]. Pain and anxiety are interdependent, forming a vicious cycle where greater pain leads to increased anxiety, intensifying the pain experience. Pain is the most frequently reported symptom of cancer, significantly impacting quality of life, nutritional status, daily functioning, emotional state, and mood [8,9]. It also serves as a prognostic factor for shorter survival in cancer patients. Effective treatment of coexisting depression and mood disorders is crucial for optimizing cancer treatment, yet cancer pain remains inadequately managed despite medical advances [10]. From the onset of diagnosis, cancer brings about negative emotions. Studies indicate that emotional experiences are critical for pathogenesis, progression, and therapy success [11]. Coping with and accepting the disease are increasingly recognized as key factors affecting quality of life and treatment success [12,13]. Stress heightens pain sensitivity, highlighting the need for behavioral and psychological interventions to manage negative emotions and teach coping strategies [14,15]. Patients need to feel empowered to influence their disease and adopt coping strategies to minimize its impact on daily life.

There is limited amounts of the literature on pain coping strategies in lung cancer and the impact of nutritional status on responses related to pain and stress [16,17,18]. Alongside diagnosis and treatment, assessing the patient’s psycho-emotional state and coping strategies is crucial for effective treatment outcomes. Patients who believe in their ability to control pain and their health, in general, show a more positive attitude, better cooperation, and more proactive health-related actions [19,20].

Understanding the patient’s approach to pain control will enable healthcare providers to ensure the expected treatment options. The study indicates discrepancies in pain coping strategies depending on the histological type of cancer. The most common pain-coping strategy in SCLC was praying or hoping, while increased behavioral activity and active coping were found in non-small-cell lung cancer (NSCLC) [21]. Another study confirmed that malnutrition is significantly more common in patients using negative strategies for coping with cancer. A lack of constructive coping is a statistically significant predictor of an increased risk for malnutrition. An advanced cancer stage is a statistically significant independent predictor of malnutrition, increasing the risk of malnutrition by more than twofold [16].

With reference to the needs of patients and their experiences of intense pain, as well as factors related to malnutrition, which significantly affect the evaluation of the quality of life, efforts were made to determine the correlation between the belief that it is possible to control pain and the strategies used to cope with it, and with malnutrition, in a group of lung cancer patients [16]. 

The relationship between pain, coping strategies, and nutritional status in lung cancer patients is a critical but underexplored area in clinical research. While the psycho-emotional state of patients and their coping mechanisms are increasingly recognized as being pivotal for therapeutic success [22], specific studies focusing on pain levels and coping strategies as determinants of malnutrition risk are sparse. The chronic nature of pain can result in a catabolic state, where the body breaks down muscle and fat stores, exacerbating malnutrition and cachexia. Despite this, there is limited research examining how varying levels of pain directly contribute to the risk of malnutrition in lung cancer patients. The complex interplay between pain, coping strategies, and nutritional status is not well-documented. While it is known that pain can heighten stress and anxiety, leading to a poorer nutritional intake, the extent to which different coping strategies can mitigate them remains unclear. Moreover, there is a need to understand how these factors collectively impact lung cancer patients’ overall prognosis and quality of life.

The present study focuses on filling an important gap in the literature, which is the lack of reports on the relationship between the assessment of the level of pain, beliefs about pain, and coping strategies as factors increasing the risk of malnutrition in lung cancer patients. We hope that the analyses conducted will allow for a detailed understanding of these relationships and contribute to developing a comprehensive care strategy and improving the overall quality of life and treatment outcomes of patients with lung cancer.

This study aims to assess the relationship between pain levels, pain-related beliefs, and coping strategies with the risk of malnutrition in lung cancer patients. Specifically, we hypothesize that higher levels of pain and negative coping strategies are associated with an increased risk of malnutrition, while positive coping strategies and effective pain management correlate with better nutritional statuses. We also aim to identify significant predictors of nutritional statuses among various demographic and clinical factors in this patient population.

## 2. Materials and Methods

### 2.1. Participants and Design

The study included 257 people diagnosed with lung cancer who gave informed consent to participate in the research. Participation was voluntary and anonymous. Each patient was informed about the purpose of the study and the possibility of withdrawal from it at any stage. The research adhered to the principles of Good Clinical Practice and complied with the ethical standards outlined in the Declaration of Helsinki. The study protocol was approved by the independent Bioethics Committee of Wroclaw Medical University, Poland (approval no. KB–729/2019).

### 2.2. Recruitment Process

Patients diagnosed with lung cancer were recruited from the oncology departments of Wroclaw Medical University. Potential participants were identified through patient records and approached during routine clinical visits. They were provided with detailed information about the study, including its purpose, procedures, and the right to withdraw at any stage. Participation was voluntary and anonymous. Informed consent was obtained from each participant before their inclusion in the study. The recruitment process ensured that all eligible patients had an equal participation opportunity.

### 2.3. Qualification Criteria

Inclusion criteria were the consent to participate in the study, an age > 18 years, a histopathologically confirmed lung cancer diagnosis, and the ability to complete the questionnaires independently. Exclusion criteria included (1) a lack of voluntary and informed consent to participate in the study, (2) an inability to complete the questionnaires independently, (3) severe pain other than that caused by the underlying disease, (4) the coexistence of other severe chronic diseases that could influence the perception of one’s health condition (another type of diagnosed malignant neoplasm, severe heart failure, severe COPD, asthma, hemodynamic instability), and (5) cognitive impairment indicative of dementia.

### 2.4. Measurement Tools

Patients qualified for the study after the inclusion and exclusion criteria were surveyed, and sociodemographic and clinical data were collected from the available medical records of the patients. Each participant was informed about the purpose and course of the study. All qualified patients gave written consent to complete the questionnaires. The overriding principle of the study was to complete the questionnaires independently in the presence of the researcher. If patients had problems completing the questionnaires themselves, the researcher assisted them. Even in such a case, the answers to the questions were those given by the patients. Only standardized tools, listed and described below, were used for the study. Some questionnaires were purchased from the Psychological Test Laboratory.

#### 2.4.1. Initial Nutrition Assessment Questionnaire

The Mini Nutritional Assessment (MNA) evaluates the nutritional status of elderly patients through anthropometric measurements, generally dietary and subjective assessments. The total score categorizes patients as well-nourished, at risk of malnutrition, or malnourished. The MNA is validated and widely used due to its reliability and ease of use (scale sensitivity—97.9%, scale specificity—100%) [23].

#### 2.4.2. Visual Analog Scale

The Visual Analog Scale (VAS) was used to assess pain intensity. It is a graphic descriptive scale using a 10 cm line on which a patient marks a point corresponding to the intensity of the experienced pain. There are 10 points on the line, where 0 means no pain, 5 means moderate pain, and 10 is unbearable pain [24].

#### 2.4.3. Beliefs about Pain Control

The Beliefs about Pain Control Questionnaire (BPCQ) is a tool used to assess patients’ beliefs regarding their pain control. It includes various domains such as the perceived influence of doctors, the self, and chance on pain control. The BPCQ is widely used in clinical settings to understand patients’ perceptions and tailor pain management strategies accordingly. The questionnaire has been validated and is known for its reliability and ease of use [25]. The adopted markings and psychometric scores for the BPCQ are presented in Table 1.

#### 2.4.4. Coping Strategies Questionnaire

The Coping Strategies Questionnaire (CSQ) by Rosenstiel and Keefe [26], containing 42 items, was used to assess pain coping methods. It includes seven subscales with six items each. Respondents rated the frequency of using each strategy on a 7-point Likert scale (0—never to 6—always). Higher scores indicate greater use of a coping strategy. Additionally, the questionnaire includes two items on perceived effectiveness: CSQ-CP (control over pain) and CSQ-AR (ability to decrease pain), both measured on a scale of 0–6 points. The adopted markings and psychometric scores for the CSQ are presented in Table 2.

### 2.5. Statistical Analysis

The statistical analysis of the survey results was performed using STATISTICA 13.3 (TIBCO Software Inc., Palo Alto, CA, USA). The assessment of the compliance of the empirical distributions of continuous quantitative variables (e.g., age) and discrete variables (survey results) with theoretical normal distributions was checked using the Kolmogorov–Smirnov–Lilliefors and Shapiro–Wilk tests. These tests were chosen to determine the normality of data distribution, which is crucial for selecting appropriate parametric or non-parametric tests. The critical value of *p* < 0.05 was assumed to be statistically significant. 

For quantitative variables, mean values (M), standard deviations (SD), medians (Me), and lower (Q1) and upper (Q3) quartiles were calculated. In the tables and figures, variables with a near-normal distribution were characterized by mean and standard deviation —M ± SD, and variables with a distribution significantly different from normal were presented as medians and quartiles—Me [Q1; Q3]. 

For qualitative variables, frequencies (*n*) and proportions (%) were calculated and collected in contingency (cross) tables. Hypotheses of no correlation between two qualitative variables were verified using Pearson’s chi-square test (*R*^2^) or Fisher’s exact test. Pearson’s chi-square test was chosen due to its ability to test the independence between categorical variables, while Fisher’s exact test was used for small sample sizes to ensure accurate *p*-values. A test result of *p* < 0.05 was taken as a significant correlation between variables. 

Linear regression and correlation analysis were used to assess the strength and nature of the relationship between two quantitative variables. Spearman’s rho rank correlation coefficients were estimated because they are suitable for non-parametric data and can assess monotonic relationships. The cut-off values of the quantitative variables separating the two states (risk of malnutrition vs. normal nutritional status) based on survey results were determined using ROC curve analysis and the value of Youden’s J statistic. ROC curve analysis was employed to determine the diagnostic ability of the test, and Youden’s J statistic was used to find the optimal cut-off point. The size of the area under the curve (AUC) was estimated for each prognostic parameter to evaluate the overall performance of the diagnostic test. 

Multivariate regression was employed to assess the significance of the effect of the analyzed predictors (independent variables) on the dependent (described) variable. The models used ridge regression, which incorporates a penalty parameter to reduce collinearity among variables, resulting in a simpler and more reliable regression function. The Mann–Whitney U test assessed the effect of qualitative predictors (sex, marital status, cancer in the family, type of cancer) on pain severity (VAS) because it is a non-parametric test suitable for comparing two independent groups. Logistic regression was utilized to evaluate the effect of qualitative predictors on the binary dependent variable. Model coefficients were estimated using the maximum likelihood method. Appropriate odds ratios with confidence intervals were calculated to assess the association’s strength and direction.

## 3. Results

### 3.1. Patient Demographics

The study included 257 patients (115 females and 142 males) aged 25–87 (mean *M* = 63, standard deviation *SD* = 9) in civil partnerships (60%). NSCLC was diagnosed in 72% of respondents. Almost 60% of the respondents underwent surgery and chemotherapy. The patients were experiencing cancer-related pain at a 5, according to the VAS. The assessment of nutritional status indicated that the median MNA questionnaire score was 22.5 in the study group [18,19,20,21,22,23,24,25,26].

### 3.2. Beliefs and Strategies for Pain Control

The analysis of beliefs about the ability to control pain showed that most patients were convinced that their pain control ability was a chance event (*Me* = 22 [21–24]) or was under the control of doctors (*Me* = 16 [11–19]). The least frequently chosen option was internal pain control (*Me* = 14 [12–16]), i.e., the ability to cope with ailments independently.

In terms of pain management strategies, the surveyed lung cancer patients most frequently chose the following: praying/hoping [18], increased behavioral activity [17], and diverting attention [17]. Patients perceived the ability to control pain and the ability to reduce pain equally effectively [3 points on a scale from 0 to 6] (Table 3).

### 3.3. Nutritional Status and Beliefs and Strategies for Pain Control Analysis

**Figure 1:**
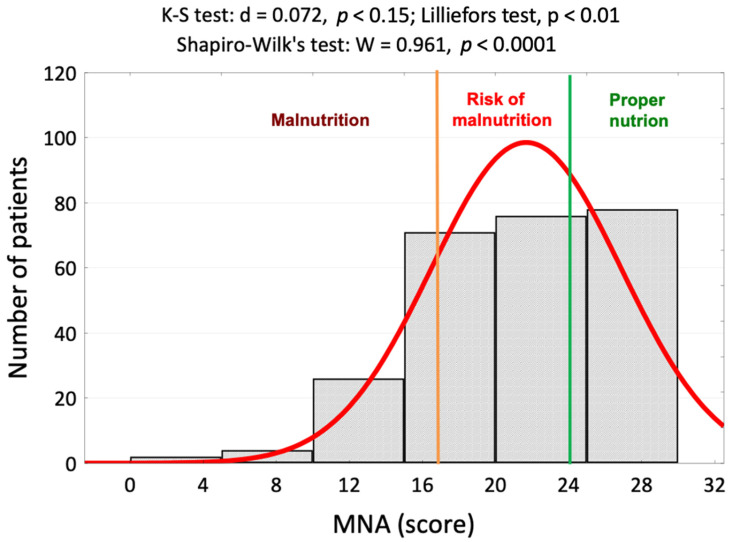
Histogram of the nutritional status assessments of 257 lung cancer patients against the background of a normal distribution and results of normality tests. Abbreviations: MNA—Mini Nutritional Assessment.

In a group of 257 lung cancer patients, a normal nutritional status was observed in 102 (39.7%), 110 (42.8%) were at risk of malnutrition, and a malnutrition status was found in 45 (17.5%). The distribution of the MNA variable deviated significantly from a normal distribution (Figure 1).

### 3.4. Correlation Analysis

Spearman’s rho rank correlation coefficient was used to assess the strength and direction of correlations with other parameters. The results of the tests are presented in Table 4.

**Figure 2:**
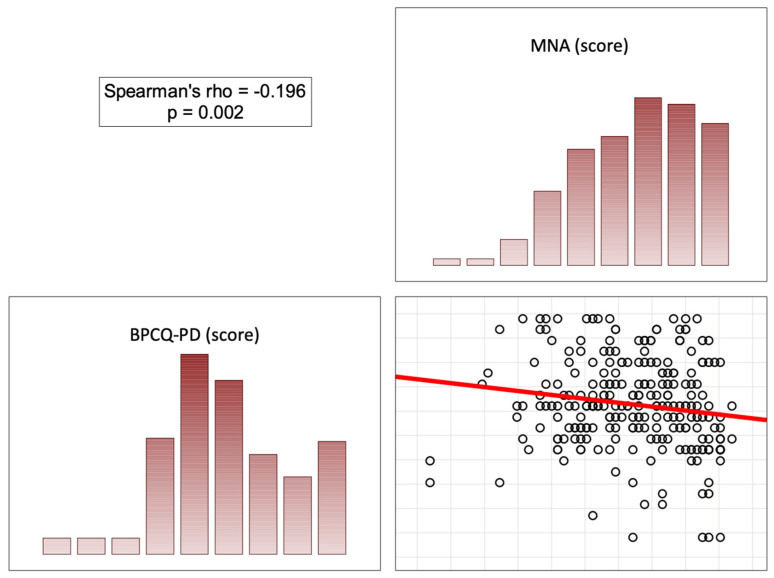
Correlation diagrams between nutritional statuses and the doctors’ influence on patients’ beliefs regarding their pain control. Abbreviations: MNA—Mini Nutritional Assessment; BPCQ—Beliefs about Pain Control Questionnaire.

**Figure 3:**
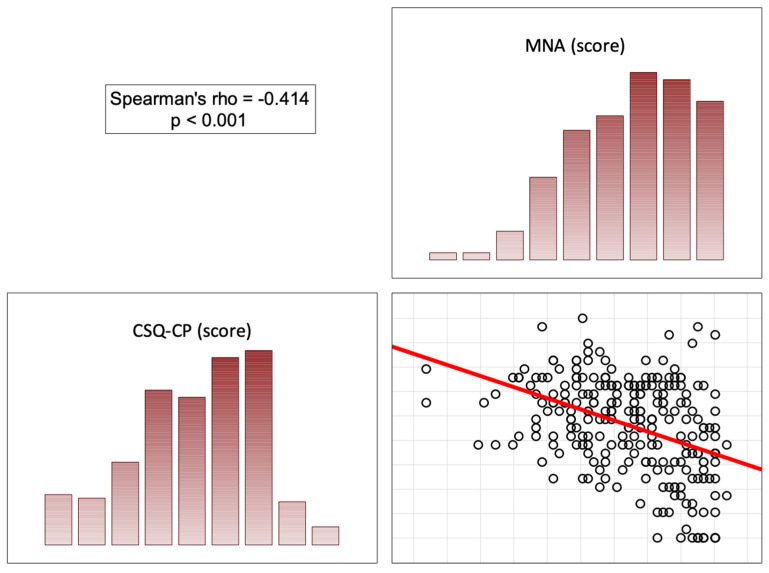
Correlation diagrams between nutritional statuses and the doctors’ influence on patients’ coping strategies (catastrophizing). Abbreviations: MNA—Mini Nutritional Assessment; CSQ-CP—Coping Strategies Questionnaire (catastrophizing).

**Figure 4:**
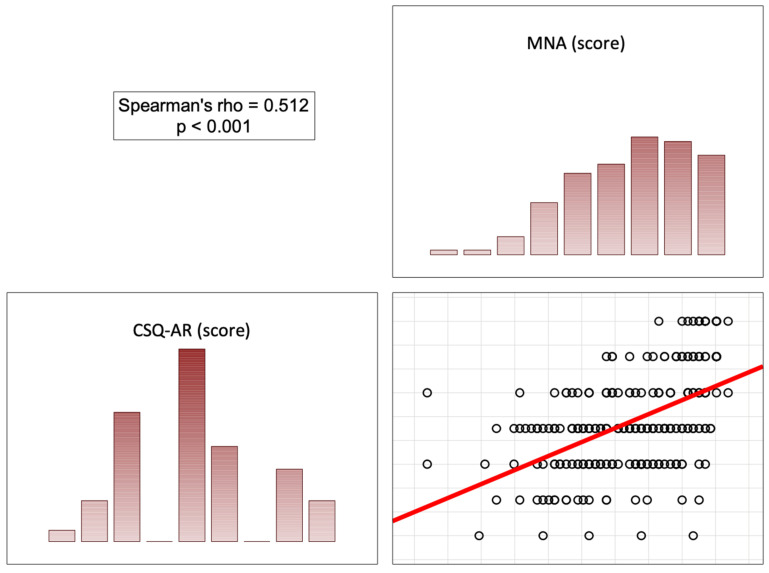
Correlation diagrams between nutritional statuses and the doctors’ influence on patients’ coping strategies (ability to reduce pain). Abbreviations: MNA—Mini Nutritional Assessment; CSQ-AR—Coping Strategies Questionnaire (ability to reduce pain).

**Figure 5:**
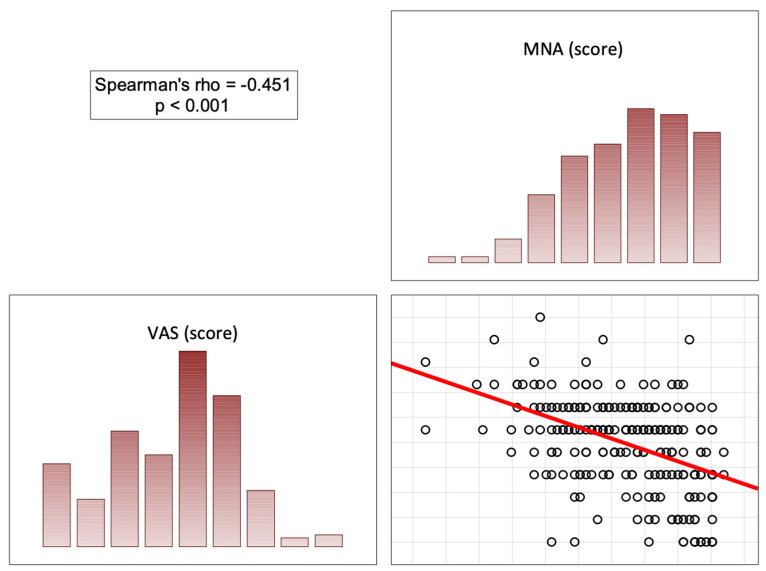
Correlation diagrams between nutritional statuses and the doctors’ influence on patients’ level of experienced pain. Abbreviations: MNA—Mini Nutritional Assessment; VAS—Visual Analog Scale.

In the opinion of the patients, the more correct the nutritional status (high scores on the MNA), the less significant the role of the doctor was in pain control (score on the BPCQ), the less frequent negative pain coping strategies were, and the more frequent positive pain control strategies and the ability to reduce pain were. In patients with a normal nutritional status, a higher score on the MNA correlated with a lower level of perceived pain on the VAS (rho = −0.451; *p* < 0.001) (Figure 2, Figure 3, Figure 4 and Figure 5).

### 3.5. Multivariate Regression Analysis

Multivariate regression was utilized to determine independent predictors of nutritional status; its results are shown in Table 5.

The results of the multivariate regression analysis between the nutritional status assessment (MNA) and pain coping strategy and pain level showed that a better nutritional status (higher MNA score) was accompanied by a younger age (β = −0.094; *p* < 0.001), NSCLC (β = 1.218; *p* = 0.037), a greater ability to reduce pain (CSQ-AR) (β = 0.901; *p* < 0.001), a lower level of pain catastrophizing (CSQ-CP) (β = −0.165; *p* = 0.001), and a lower level of perceived pain (VAS) (β = 0.639; *p* < 0.001). The mathematical model of the nutritional assessment (MNA) as a function of age, cancer type, pain level (VAS), and pain coping strategies adopted in the CSQ takes the following form:

MNA = 28.9 − 0.09 × Age + 1.19 × NSCLC + 0.95 × CSQ-AR − 0.17 × CSQ-CP − 0.65 × VAS ± 4.1

The model as a whole is adequate (*F* = 30.6, *p* < 0.001) and explains approximately 37% of the variability in MNA scores (coefficient of determination *R*^2^ = 0.366).

### 3.6. Logistic Regression Analysis

**Figure 6:**
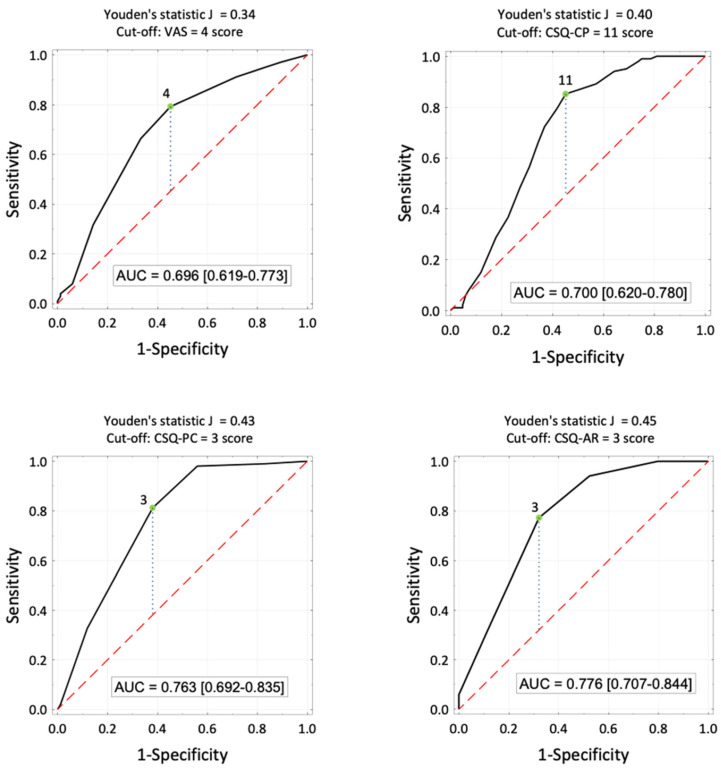
ROC curves for estimating the probability of malnutrition based on the level of pain, catastrophizing (stimulators), pain control, and the ability to reduce pain (destimulators), the proposed cut-off values and areas under the ROC curves, and their 95% confidence intervals.

Logistic regression was employed to predict malnutrition or its risk based on BPCQ, CSQ, and VAS scores. Patients with an MNA score of <23 were classified as at risk of malnutrition (*Y* = 1). Quantitative variables were transformed into binary variables based on cut-off values determined by the ROC curve analysis (Figure 6). The results of the ROC curve analysis are shown in Table 6.

The chance of malnutrition in patients experiencing pain levels of 4 or more is almost six times higher (OR = 5.77) compared to patients experiencing pain levels of less than 4. The chance of malnutrition in patients using catastrophizing (CSQ-CP) with 11 or more points as a pain coping method is eight times higher (OR = 8.08) compared to patients with CSQ-CP scores < 11. The chance of malnutrition in patients with pain control scores of up to 3 points is seven and a half times higher (OR = 7.49) than in patients with CSQ-PC scores > 3. The chance of malnutrition in patients with a pain reduction ability score of up to 3 is seven times higher (OR = 7.02) compared to patients with CSQ-AR scores > 3.

Due to the correlations between the descriptor variables, multivariate logistic regression analysis was utilized to establish independent predictors of malnutrition risk. Its results are presented in Table 7.

The model for predicting malnutrition or malnutrition risk from the BPCQ, CSQ, and VAS takes the following logit form:

logit *P*{*Y* = 1|*X*} = −2.44 + 1.55 × (CSQ-CP ≥ 11) + 0.98 × (CSQ-AR ≥ 3) + 1.14 × (VAS ≥ 4)

The model is significant as a whole (R^2^ = 96.3, *df* = 3, *p* < 0.0001), and all its coefficients are significant at *p* < 0.05.

The probability of malnutrition in lung cancer patients increases after exceeding the score of 11 in coping with pain by catastrophizing (CSQ-CP ≥ 11), with a score in the ability to reduce pain of less than 4 (CSQ-AR ≥ 3), and with a level of perceived pain (VAS) ≥ 4.

## 4. Discussion

Our study indicated that more than half of lung cancer patients have an abnormal nutritional status. The leading causes of malnutrition include an insufficient calorie intake, impaired metabolism, fatigue, depression, and treatment-related toxicity. Reports in the literature demonstrate that patients with lung cancer are a group at risk of malnutrition, and many are diagnosed with nutrient deficiencies and cachexia [27]. Malnutrition negatively affects treatment options and disease prognosis, with an unfavorable prognosis of higher mortality risk [28]. In the case of cancer patients, both the malignancy itself and the treatment have a significant impact on the progression of malnutrition and cachexia. Often, malnutrition is diagnosed in this group of patients even before treatment starts [29,30,31].

The findings of our study align with and expand upon the results reported by Nardi et al. [32], who assessed the association between malnutrition and anxiety in hospitalized adult cancer patients. Their study found significant links between higher Nutrition Risk Screening (NRS) scores, disease stage, cachexia, and increased anxiety symptoms. Additionally, they noted that cancer sites other than those that are gastrointestinal involved a lower risk of anxiety symptoms. Nardi et al. emphasized the need for routine nutritional assessments and mental health screenings for cancer patients to mitigate the dual burden of malnutrition and anxiety. Our findings support this recommendation and further suggest that comprehensive pain management strategies incorporating pharmacological and behavioral approaches are essential.

Bagan et al. [29] found that malnutrition is a predictor of major postoperative complications, with an odds ratio of 1.76 and 90-day mortality with an OR of 6.5 in patients undergoing pneumonectomy for NSCLC. In another study, malnutrition significantly affected the 5-year survival rate. An abnormal body weight may inhibit the response to treatment [33]. Both cancer and cancer treatments affect patients’ nutritional status due to a lower food intake and an altered metabolism [34].

Furthermore, the molecular mechanisms underlying pain and malnutrition in lung cancer are complex and interconnected. Key factors include the nerve growth factor (NGF) and its tropomyosin receptor kinase A (TrkA), prostaglandins, inflammatory cytokines, transient receptor potential vanilloid 1 (TRPV1), and endogenous opioids. NGF and TrkA are crucial for the growth and survival of neurons and cancer cells, contributing to pain through neural sensitization [35]. Prostaglandins and inflammatory cytokines are mediators of inflammation that can exacerbate pain and are often elevated in cancer. TRPV1 is involved in pain perception and is often upregulated in cancerous tissues [36]. Additionally, endogenous opioids, which typically modulate pain, can be dysregulated in cancer, leading to inadequate pain control. Malnutrition and cachexia, frequently observed in cancer patients, further complicate the situation by exacerbating inflammation and pain through these molecular pathways [37].

Somatic complaints and pain symptoms are significant problems found in lung cancer patients. The psychological aspect is also one of the main problems of a lung cancer patient with pain. Patients experience anxiety, fear, and distress, which can develop into frustration, sadness, and depression. It is important to note that such long-lasting emotional states can result from pain and be its cause. In the case of chronic pain, it is appropriate to speak of a mental vicious cycle [38].

Chabowski et al. [17] reported that the level of anxiety, depression, and perceived pain differed significantly between patients with a normal nutritional status and those at risk of malnutrition and between patients with a normal nutritional status and those who are malnourished (all *p* < 0.001). Differences between malnourished patients and those at risk of malnutrition were insignificant.

This means that the molecular mechanisms of pain and malnutrition are directly linked to the broader discussion about the impact of cancer and its treatments on nutritional status and pain in lung cancer patients.

Very often, an abnormal nutritional status is associated with more severe complaints. Patients with lung cancer at risk of or with an abnormal body weight are more likely to report a higher severity of disease symptoms and a greater severity of mood disorders. Chabowski et al. demonstrated that malnourished lung cancer patients experience more severe somatic symptoms, pain, anxiety, and depression than patients with normal weights [17]. Additionally, depression, along with other factors, was significantly associated with more pain-related interference [39]. In another study, severe pain was identified as a risk factor for increased postoperative depression in patients with NSCLC [40]. In the same study, a statistically significant correlation was reported between malnutrition, depression, and anxiety [39].

Few studies simultaneously address coping with pain, strategies, and malnutrition [16,18,41]. Some studies describe coping strategies for cancer or pain in correlation with the quality of life [18]; others describe malnutrition in correlation with the quality of life [42,43,44,45]. Our study brings new insights into the status of coping with pain and malnutrition among lung cancer patients.

Coping with chronic pain and how the patient controls it is an increasingly common problem. It is influenced by internal-psychological, external-social, and disease-related factors. Thus, the attitude toward the experienced pain adopted by a person will depend on many variables, which, in turn, leads to undertaking specific pain coping strategies [46]. In light of the above, it is worth emphasizing the importance of assessing nutritional statuses and diagnosing malnutrition as a factor related to pain, coping strategies, and the sense of control over pain, which is linked with the sense of subjectivity and the ability to influence one’s own sensations and environment [8].

Polański et al. [47] analyzed the pain coping strategies according to the histological type of lung cancer. The results indicated that patients chose strategies according to cancer type, and among those with SCLC, negative strategies (praying or hoping) were more frequent. Among patients with NSCLC, positive strategies (increased behavioral activity and active coping) were more common.

However, the above study did not consider nutritional status (malnutrition), which is related to the severity of pain and the type of pain coping strategies employed. Our present study showed that the doctor’s role in pain control is reduced in patients with normal nutritional statuses, and negative pain coping strategies are used less frequently, while positive ones are more common. A normal nutritional status correlates with lower levels of perceived pain. The chance of malnutrition in patients with low pain control scores and low pain reduction ability is seven and a half times higher compared to patients with higher scores.

In ours and other studies, a better nutritional status was linked with lower levels of pain, confidence in the ability to reduce and manage pain on one’s own, and less frequent use of coping strategies based on catastrophizing. Our results confirm that the likelihood of malnutrition in patients who use catastrophizing to cope with pain is much higher than in patients who use positive methods to do so.

Research on 310 lung cancer patients documented that nutritional status was a significant predictor of quality of life across all domains of the QOL-C30. A better nutritional status was associated with better scores across all domains of functioning and a lower severity of cancer-related complaints and their impact on quality of life [41]. Nutritional status is, therefore, a factor that significantly impacts many aspects of normal functioning.

One more interesting observation of our research is the correlation between nutritional status and the domain of the BPCQ (the power of doctors). People with a sense of external control believe that the illness is imposed on them; they are convinced that it is impossible to change their situation, and the reluctance to take action to reduce pain is predominant. It is difficult for such a person to establish contact with a doctor, making recovery difficult. This attitude promotes general dissatisfaction and negative emotions [48]. The literature reports that the choice of pain control strategy may depend on the causes of pain [48]. Patients whose pain is under some personal control (e.g., conditions in which active muscle work can reduce pain) attribute a lesser role to external factors in pain. In contrast, patients with cancer or migraines feel a greater influence of external factors on pain control.

Identifying factors contributing to a worsening nutritional status in cancer patients is essential for planning treatment [49]. Most importantly, our study brings some insights and points to the urgent need for behavioral interventions in addition to standard pharmacological and surgical treatment. Payne et al. [50] suggest that interventions to improve patients’ nutritional statuses may benefit physical strength and functional capacity. However, all interventions should be undertaken with utmost caution due to the lack of compelling evidence.

### 4.1. Study Limitations

The limitations of this study include the recall bias arising from the use of self-reported questionnaires, as these rely on the participants’ perceptions and may be subject to response biases. Additionally, the absence of a control group limits the ability to compare findings directly with non-cancer populations or patients with different types of cancer. Consequently, while we could identify correlations between pain coping strategies, pain levels, and malnutrition within lung cancer patients, the generalizability of these findings to other populations remains uncertain. These limitations suggest that future studies should consider incorporating control groups and alternative methods of data collection, such as objective measures of nutritional status and pain, to validate and extend our findings. While this study offers valuable insights into the relationship between pain, coping strategies, and malnutrition in lung cancer patients, caution should be exercised in generalizing these results. Future research should aim to include control groups and utilize objective assessment tools to enhance the robustness and applicability of the findings.

### 4.2. Practical Implications

The primary approach to preventing malnutrition in lung cancer patients is early identification and the regular assessment of nutritional status. Implementing routine nutritional screenings can help healthcare providers detect and address malnutrition at an early stage. Personalized interventions, including dietary modifications and behavioral therapy, are essential in helping patients manage their pain and improve their nutritional status. Effective pain management is another critical aspect of treatment planning. Comprehensive pain assessments should be conducted regularly, and pain management strategies should incorporate pharmacological and behavioral approaches. Educating patients on effective pain coping strategies and providing psychological support can enhance their ability to manage pain and reduce the risk of malnutrition. Furthermore, integrating individualized treatment plans that consider the patient’s age, cancer type, and coping mechanisms can improve overall patient care and quality of life. Such a personalized approach allows for more targeted and effective interventions, addressing each patient’s unique needs. There is a need for further research to explore the intricate relationship between nutritional status, pain, and pain coping strategies in lung cancer patients. Future studies should aim to provide a more in-depth understanding of these mechanisms and evaluate the effectiveness of different therapeutic interventions to enhance patient care. By incorporating these practices into clinical settings, healthcare providers can better manage the multifaceted challenges associated with lung cancer, ultimately improving patient outcomes and their quality of life.

## 5. Conclusions

This study reveals that only 40% of lung cancer patients maintain a normal nutritional status. Our findings indicate that a better nutritional status is associated with a younger age, NSCLC diagnosis, lower pain levels, a greater ability to reduce pain, and lower scores in pain coping domains based on catastrophizing. The risk of malnutrition in patients experiencing moderate to severe pain is almost six times higher than that of those with less intense pain. Similarly, the likelihood of malnutrition is eight times higher in patients who use catastrophizing to cope with pain. Moreover, patients with low pain control scores and low pain reduction abilities are seven and a half times more likely to suffer from malnutrition. A normal nutritional status correlates with a reduced reliance on doctors for pain control, a decreased use of negative coping strategies, and an increased use of positive coping mechanisms. These findings underscore the complex interplay between nutritional status, pain, and coping strategies in lung cancer patients.

## Figures and Tables

**Table 1:** Psychometric assessment of the BPCQ questionnaire.

BPCQ Subscale	*α*	*r*
BPCQ-IF (internal factors)	0.736	0.364
BPCQ-PD (power of doctors)	0.812	0.487
CSQ-CP (chance events)	0.653	0.324

Abbreviations: *α*—Cronbach’s alpha, *r*—average correlation between items and scale.

**Table 2:** Psychometric assessment of the CSQ questionnaire.

CSQ Subscale	*α*	*r*
CSQ-DA (diverting attention)	0.846	0.487
CSQ-RP (reinterpreting pain sensation)	0.812	0.487
CSQ-CP (catastrophizing)	0.759	0.346
CSQ-IP (ignoring pain sensation)	0.837	0.465
CSQ-PH (praying and hoping)	0.815	0.443
CSQ-CS (coping self-statements)	0.824	0.442
CSQ-IB (increased behavioral activity)	0.860	0.512

Abbreviations: *α*—Cronbach’s alpha, *r*—average correlation between items and scale.

**Table 3:** Characteristics of 257 patients treated for lung cancer.

Characteristic	Statistics
Male, *n* (%)	142 (55.3)
Age (years), *M* ± *SD*	63.2 ± 9.4
Marriage or civil partnership, *n* (%)	154 (59.9)
Cancer in the family, *n* (%)	98 (38.1)
Non-small-cell lung cancer, *n* (%)	185 (72.0)
Treatment	
Surgery, *n* (%)	149 (58.0)
Radiotherapy, *n* (%)	82 (31.9)
Chemotherapy, *n* (%)	164 (63.8)
Symptomatic treatment, *n* (%)	35 (13.6)
Complementary unconventional treatment, *n* (%)	7 (2.7)
VAS (score), *Me* [*Q*1–*Q*3]	5 [3–6]
MNA (score), *Me* [*Q*1–*Q*3]	22.5 [18–26]
**BPCQ**	
Internal factors BPCQ-IF (score), *Me* [*Q*1–*Q*3]	14 [12–16]
Power of doctors BPCQ-PD (score), *Me* [*Q*1–*Q*3]	16 [13–19]
Chance events BPCQ-CE (score), *Me* [*Q*1–*Q*3]	22 [21–24]
**CSQ**	
Diverting attention CSQ-DA (score), *Me* [*Q*1–*Q*3]	17 [11–21]
Reinterpretation of pain CSQ-RP (score), *Me* [*Q*1–*Q*3]	14 [9–17]
Catastrophizing CSQ-CP (score), *Me* [*Q*1–*Q*3]	14 [10–18]
Ignoring pain CSQ-IP (score), *Me* [*Q*1–*Q*3]	14 [9–17]
Praying/hoping CSQ-PH (score), *Me* [*Q*1–*Q*3]	18 [14–22]
Coping self-statements CSQ-CS (score), *Me* [*Q*1–*Q*3]	16 [12–20]
Increased behavioral activity CSQ-IB (score), *Me* [*Q*1–*Q*3]	17 [12–21]
Pain control CSQ-PC (score), *Me* [*Q*1–*Q*3]	3 [2–4]
Ability to reduce pain CSQ-AR (score), *Me* [*Q*1–*Q*3]	3 [2–4]

Abbreviations: *M* ± *SD*—mean ± standard deviation, *Me* [*Q*1–*Q*3]—median and quartiles, *n* (%)—number and percentage; VAS—Visual Analog Scale; MNA—Mini Nutritional Assessment; BPCQ—Beliefs about Pain Control Questionnaire; CSQ—Coping Strategies Questionnaire.

**Table 4:** Values of Spearman’s rank correlation coefficients between the nutritional statuses on the MNA and pain coping strategies.

Domains of Pain Coping Strategies	Spearman’s Correlation	
	rho	*p*-Value
BPCQ-IF (internal factors)	0.087	0.165
BPCQ-PD (power of doctors)	−0.196	0.002
BPCQ-CE (chance events)	0.085	0.173
CSQ-DA (diverting attention)	−0.031	0.620
CSQ-RP (reinterpretation of pain)	−0.194	0.002
CSQ-CP (catastrophizing)	−0.414	<0.001
CSQ-IP (ignoring pain)	−0.198	0.001
CSQ-PH (praying/hoping)	−0.253	<0.001
CSQ-CS (coping self-statements)	−0.172	0.006
CSQ-IB (increased behavioral activity)	0.081	0.195
CSQ-PC (pain control)	0.499	<0.001
CSQ-AR (ability to reduce pain)	0.512	<0.001
Pain perceived on the VAS	−0.451	<0.001

Abbreviations: BPCQ—Beliefs about Pain Control Questionnaire; CSQ—Coping Strategies Questionnaire; VAS—Visual Analog Scale.

**Table 5:** Results of univariate and multivariate regression analysis on relationships between the nutritional status assessment (MNA), pain coping strategies, and pain levels.

Predictors of a Good Nutritional Status	Univariate Analysis		Multivariate Analysis	
	β	*p*	β	*p*
BPCQ-PD (power of doctors) score	−0.191	0.013	-	-
CSQ-RP (reinterpretation of pain) score	−0.161	0.003	-	-
CSQ-CP (catastrophizing) score	−0.370	<0.001	−0.165	0.001
CSQ-IP (ignoring pain) score	−0.146	0.006	-	-
CSQ-PH (praying/hoping) score	−0.191	<0.001	-	-
CSQ-CS (coping self-statements) score	−0.144	0.006	-	-
CSQ-PC (pain control) score	1.994	<0.001	-	-
CSQ-AR (ability to reduce pain) score	1.895	<0.001	0.901	<0.001
Pain perceived on the VAS (score)	−1.184	<0.001	−0.639	<0.001
Age (years)	−0.116	0.001	−0.094	<0.001
Cancer in the family (yes)	−2.218	0.001	-	-
Non-small-cell lung cancer (yes)	3.051	<0.001	1.218	0.037

Abbreviations: MNA—Mini Nutritional Assessment; BPCQ—Beliefs about Pain Control Questionnaire; CSQ—Coping Strategies Questionnaire.

**Table 6:** Results of the ROC curve analysis (cut-off values and areas under the ROC curve), the number (proportion) of patients in groups with different malnutrition risks, and the significance of test results.

Predictors of Malnutrition or Risk of Malnutrition	ROC Curve Analysis		Malnutrition or Risk of Malnutrition		*p*-Value
	Cut-Off (Score)	AUC	Yes *N* = 155 (%)	No *N* = 102 (%)	
BPCQ-IF (internal factors)	≥14	0.577	86 (55.5)	44 (43.1)	0.057
BPCQ-PD (power of doctors)	≥14	0.630	127 (81.9)	61 (59.8)	<0.001
BPCQ-CE (chance events)	≥23	0.561	107 (69.0)	66 (64.7)	0.498
CSQ-DA (diverting attention)	≥7	0.531	139 (89.7)	87 (85.3)	0.330
CSQ-RP (reinterpretation of pain)	≥14	0.595	98 (63.2)	41 (40.2)	<0.001
CSQ-CP (catastrophizing)	≥11	0.700	134 (86.5)	45 (44.1)	<0.001
CSQ-IP (ignoring pain)	≥13	0.593	105 (67.7)	48 (47.1)	0.001
CSQ-PH (praying/hoping)	≥17	0.662	107 (69.0)	43 (42.2)	<0.001
CSQ-CS (coping self-statements)	≥16	0.595	92 (59.4)	42 (41.2)	0.005
CSQ-IB (increased behavioral activity)	≥20	0.549	121 (78.1)	66 (64.7)	0.022
CSQ-PC (pain control)	≥3	0.763	131 (84.2)	43 (42.2)	<0.001
CSQ-AR (ability to reduce pain)	≥3	0.776	126 (81.3)	39 (38.2)	<0.001
Pain perceived on the VAS	≥4	0.696	128 (82.6)	46 (45.1)	<0.001

Abbreviations: BPCQ—Beliefs about Pain Control Questionnaire; CSQ—Coping Strategies Questionnaire.

**Table 7:** Results of the univariate and multivariate logistic regression analysis between the risk of malnutrition and the results of assessments of pain coping strategies and the level of perceived pain.

Predictors	Univariate Analysis			Multivariate Analysis		
	β	*p*	OR (95% CI)	β	*p*	OR (95% CI)
BPCQ-PD ≤ 14	1.115	<0.001	3.05 (1.72–5.40)	−0.045	0.913	0.96 (0.43–2.14)
CSQ-RP ≥ 14	0.939	<0.001	2.56 (1.53–4.28)	−0.019	0.967	0.98 (0.40–2.41)
CSQ-CP ≥ 11	2.090	<0.001	8.08 (4.41–14.8)	1.550	<0.001	4.71 (2.40–9.26)
CSQ-IP ≥ 13	0.860	0.001	2.36 (1.41–3.96)	0.754	0.084	2.13 (0.90–5.00)
CSQ-PH ≥ 17	1.118	<0.001	3.06 (1.81–5.16)	0.278	0.471	1.32 (0.62–2.82)
CSQ-CS ≥ 16	0.735	0.005	2.09 (1.25–3.48)	−0.330	0.467	0.72 (0.29–1.76)
CSQ-PC ≥ 3	2.013	<0.001	7.49 (4.15–13.5)	0.674	0.159	1.96 (0.77–5.02)
CSQ-AR ≥ 3	1.949	<0.001	7.02 (3.97–12.4)	0.976	0.035	2.65 (1.07–6.57)
VAS ≥ 4	1.753	<0.001	5.77 (3.26–10.2)	1.143	0.001	3.14 (1.61–6.10)
Cancer in the family	1.088	<0.001	2.97 (1.70–5.19)	0.459	0.169	1.58 (0.82–3.05)
NSCLC (yes)	−0.914	0.003	0.40 (0.22–0.74)	−0.349	0.350	0.71 (0.34–1.47)

## Data Availability

The data presented in this study are available on request from the corresponding author due to legal and ethical reasons.
